# Surgical retrieval and repositioning of malpositioned prosthetic pulmonic valve in a patient with situs inversus dextrocardia and tetralogy of Fallot: a case report

**DOI:** 10.1097/RC9.0000000000000250

**Published:** 2026-02-10

**Authors:** Chusak Nudaeng, Thukten Chophel, Raschareeya Santisevi

**Affiliations:** a Institute of Cardiovascular Disease, Cardiovascular and Thoracic Surgery unit, Department of Surgery, Rajavithi Hospital, College of Medicine, Rangsit University, Bangkok, Thailand; b Department of Surgery, Jigme Dorji Wangchuck National Referral Hospital, Thimphu, Bhutan

**Keywords:** beating-heart surgery, case report, dextrocardia, MyVal valve, percutaneous pulmonary valve implantation, situs inversus, tetralogy of Fallot, valve malposition

## Abstract

**Introduction:**

Percutaneous pulmonary valve implantation (PPVI) offers a minimally invasive alternative to surgical valve replacement in patients with right ventricular outflow tract dysfunction. However, valve malposition or migration can occur, necessitating surgical retrieval.

**Case presentation:**

We report a 16-year-old male with situs inversus dextroposition, mirror-image dextrocardia, and repaired Tetralogy of Fallot (TOF), who developed severe pulmonary regurgitation and underwent PPVI using a MyVal valve in another hospital. Post-procedural imaging revealed valve malposition at the right pulmonary artery bifurcation with paravalvular leakage. Surgical retrieval and reimplantation of the same prosthetic valve were successfully performed on a beating heart under cardiopulmonary bypass.

**Discussion:**

This case highlights the challenges of percutaneous interventions in complex congenital heart anatomies and the role of early surgical management in salvaging malpositioned valves. Beating-heart reimplantation minimized myocardial ischemia and facilitated rapid recovery.

**Conclusion:**

Prompt surgical retrieval and reimplantation of malpositioned PPVs can preserve valve function, reduce morbidity, and optimize outcomes in complex congenital heart disease.

## Introduction

Percutaneous pulmonary valve implantation (PPVI) has emerged as a minimally invasive alternative to open surgical valve replacement and has demonstrated comparable efficacy in both pediatric and adult populations^[1]^. The principal advantage is its ability to avoid the risks and recovery associated with open-heart surgery^[2]^. However, procedural complications such as valve malposition, dislodgement, and migration may occur, and once deployed, the valve cannot be re-sheathed or repositioned percutaneously^[3]^. The management of complex congenital heart diseases presents significant challenges, even in cases with situs solitus levocardia. Herein, we present a rare and complex case with situs inversus dextroposition and mirror-image dextrocardia with TOF, who underwent total correction at 1 year of age. He subsequently developed severe pulmonic valve regurgitation necessitating PPVI. Although the initial implantation appeared successful, post-procedural echocardiography revealed malpositioning of the prosthetic pulmonic valve (PPV), leading to further surgical intervention.

**HIGHLIGHTS**

- Rare case of *situs inversus dextrocardia* with repaired *Tetralogy of Fallot*.
- *Percutaneous pulmonary valve* (MyVal) implantation complicated by malposition.
- *Beating-heart surgical retrieval and reimplantation* preserved valve integrity.
- Successful *reconstruction of main pulmonary artery* using bovine pericardial patch.
- One-year follow-up showed *excellent valve function and clinical recovery*.

## Methodology

This case is being reported in line with the SCARE guidelines^[4]^. Informed written consent to write this case report was obtained from the patient’s parents after clearly explaining that his identity would be deidentified.

## Case presentation

**Figure 1.**
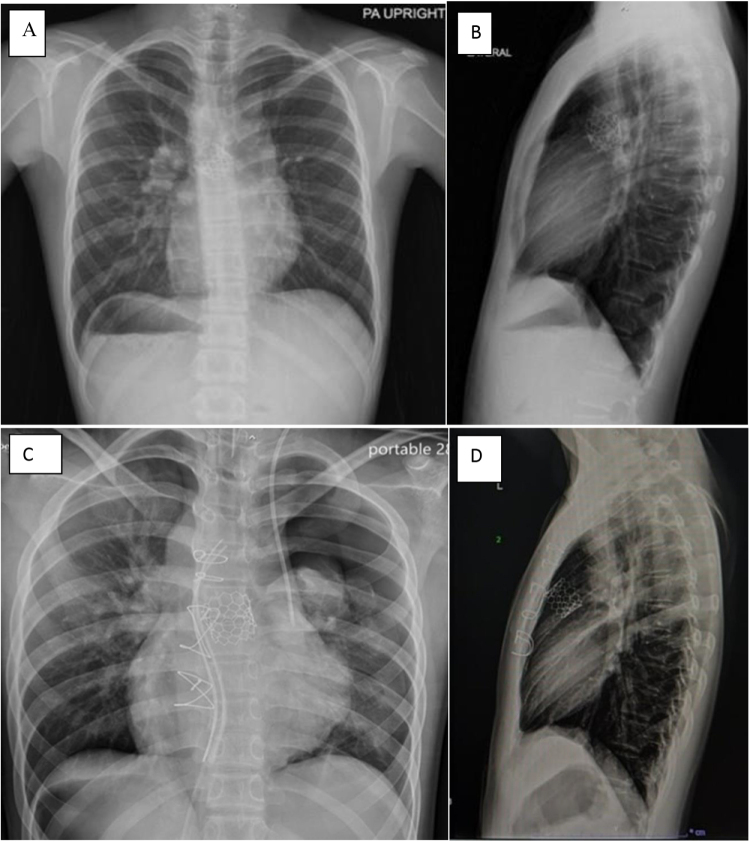
Chest X-ray PA (A) and lateral views (B) post placement of the MyVal valve percutaneously. Chest X-ray PA (C) and lateral views (D) post repositioning surgically.

A 16-year-old male is a known case of situs inversus dextroposition, mirror-image dextrocardia, and TOF with right sided aortic arch, as well as glucose-6-phosphate dehydrogenase deficiency. He had previously undergone total correction of TOF with a transannular patch repair of main pulmonary artery (MPA) at 1 year of age. In this visit, he had presented to another hospital with an exertional dyspnea and fatigue. Transthoracic echocardiography (TTE) revealed severe pulmonic valve regurgitation with a dilated right ventricle and preserved systolic function. Given the patient’s high-risk surgical history, they have decided to perform PPVI. A Myval transcatheter heart valve from Meril Life Sciences Pvt. Ltd., India, was implanted (Fig. 1A and B). On post procedure day 5, the patient was evaluated for chest discomfort with an echocardiography which raised concern for malposition of the prosthetic valve at the proximal right pulmonary artery (RPA). Despite appearing functionally competent, significant paravalvular leakage was observed, with a right ventricular outflow tract (RVOT) pressure gradient of 25–30 mm Hg and a paravalvular leak gradient of 6 mm Hg. The patient was then referred to our hospital for evaluation and surgical intervention.

**Figure 2.**
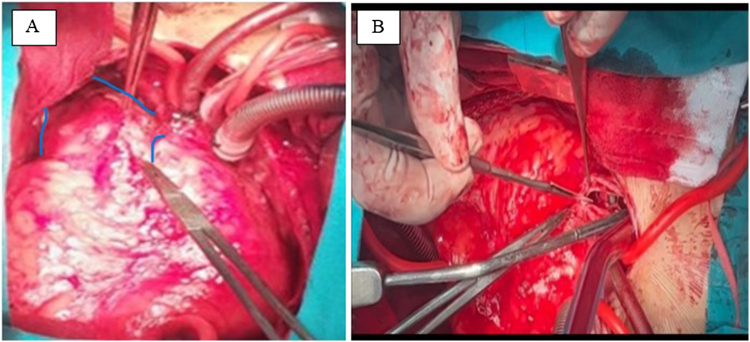
(A) Heavily calcified trans-annular patch with the PA anatomy marked in blue lines. The PPV was lodged at the confluence of RPA & LPA marked by the artery forceps. (B) Incision on MPA and excision of the calcified trans-annular patch.

**Figure 3.**
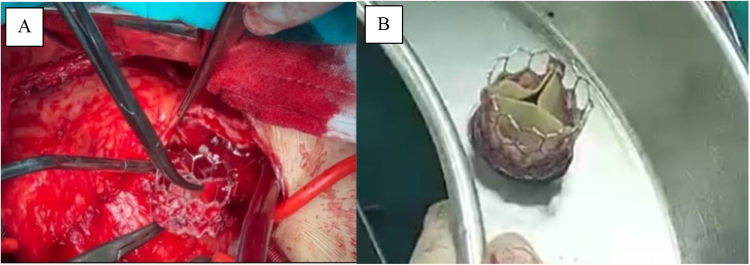
(A) Retrieval of the malpositioned MyVal valve. (B) After the salvage and cleaning.

**Figure 4.**
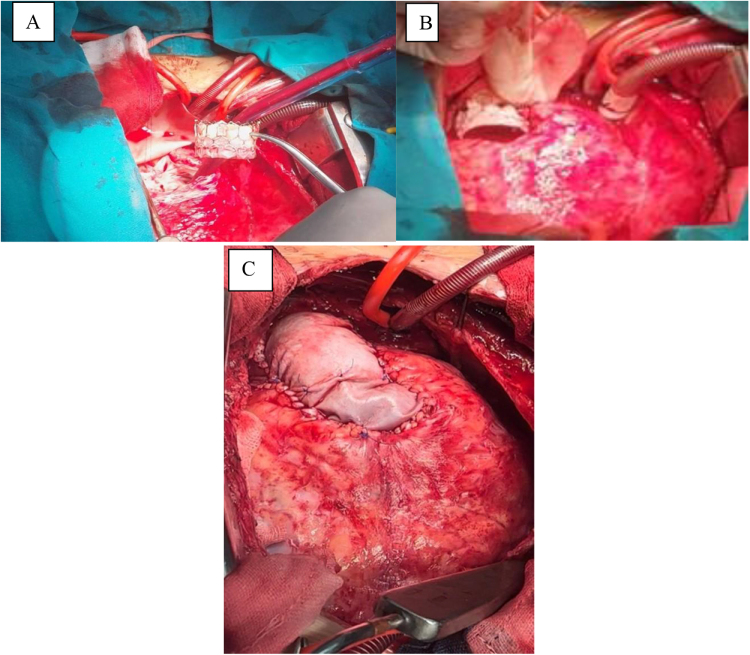
(A–C) Surgical repositioning and fixation of the MyVal valve on the pulmonary valve annulus reinforced with bovine pericardium.

Although the patient was hemodynamically stable, the clinical concern prompted an urgent decision to perform open surgical retrieval and repositioning of the PPV. After obtaining written informed consent from the parents, the patient underwent reoperative median sternotomy. Extensive adhesiolysis was performed to delineate the anatomy. The procedure was conducted on a beating heart under full cardiopulmonary bypass. The MPA was opened, revealing a heavily calcified transannular patch, which was excised (Fig. 2A and B). The mispositioned valve was found lodged at the bifurcation of the right and left pulmonary arteries and was retrieved carefully to prevent trauma to the vessel walls and valve structure (Fig. 3A). The valve was then gently cleaned with saline irrigation with care not to damage it (Fig. 3B). It was then reimplanted at the native pulmonic valve annulus and secured with 5-0 Prolene sutures at four quadrants (Fig. 4A and B). The MPA was reconstructed with a bovine pericardial patch to accommodate the valve and ensure unobstructed outflow (Fig. 4C).

The patient was weaned from bypass uneventfully. The intraoperative transesophageal echocardiogram (TEE) confirmed optimal positioning of the valve, absence of paravalvular leak, and preserved tricuspid valve function. It was reconfirmed by the postoperative chest X-ray (Fig. 1C and D). He was extubated within the first hour post-surgery and on postoperative day 1, the vitals were stable with no inotropic support.

Follow-up echocardiogram on postoperative day 2 confirmed stable valve function and no significant regurgitation or obstruction (mean pressure gradient of 4 mm Hg). The patient was discharged on postoperative day 5 with uneventful postoperative course. At the 1-week outpatient follow-up, he remained clinically well, and chest X-ray confirmed appropriate valve position. At 1 year follow up, the echocardiogram showed well positioned MyVal valve with mean pressure gradient of 8 mm Hg with mild pulmonary regurgitation and tricuspid pressure gradient of 18 mm Hg. The LV ejection fraction was 55% with fair RV systolic function and Tricuspid Annular Plane Systolic Excursion (TAPSE) of 10.1 mm. His symptoms were well controlled.

## Discussion

TOF is among the most common congenital heart defects. However, its association with dextrocardia, particularly in the context of situs inversus dextroposition and mirror-image anatomy, remains relatively rare^[5]^. Such complex anatomical variations present significant challenges for both interventional cardiologists and surgeons, particularly during procedures that rely on conventional anatomical landmarks.

The management of congenital heart disease often requires multiple staged interventions throughout a patient’s life. While percutaneous procedures like PPVI offer the advantage of reduced invasiveness, they can be associated with significant complications^[2]^. Mirror-image dextroposition is known to provide a lot of technical challenges to interventional cardiologists^[6]^. Therefore, an effective management in these cases requires close collaboration between the interventionalist and surgical teams, especially when dealing with both predictable and unpredictable procedural complications^[7]^.

PPVI may lead to a variety of intra- and post-procedural complications, including coronary artery compression, valve embolization, malposition, and pulmonary artery occlusion^[8]^. Valve migration into the RVOT is particularly dangerous and may require urgent surgical intervention to prevent life-threatening consequences^[9]^. Additionally, given the high cost of prosthetic valves, any damage during retrieval not only results in a financial burden but may also negatively impact clinical outcomes.

In the case presented, early surgical retrieval and salvage of the malpositioned prosthetic valve allowed for repositioning and successful reimplantation. This required a comprehensive understanding of the patient’s complex cardiac anatomy and meticulous surgical technique. The removal of adherent thrombus from the prosthetic valve without damaging the device was key in preserving its structural and functional integrity.

Importantly, the open surgical procedure was performed on a beating heart under cardiopulmonary bypass, thereby avoiding the myocardial stress associated with cardioplegia and reperfusion injury. Beating-heart surgery offers distinct advantages in selected patients, including reduced myocardial ischemia, improved early recovery, and preserved right ventricular function – especially beneficial in patients with right ventricle dysfunction or previous surgical repairs^[10]^.

## Conclusion

Early surgical intervention for complications such as malposition, dislodgement, or migration of prosthetic pulmonic valves during PPVI is critical for optimizing outcomes. Prompt open retrieval with careful handling of the valve, including gentle clot removal, can preserve its integrity and allow for successful reimplantation in the same surgical setting. This approach enhances postoperative hemodynamics and expedites recovery, especially in complex congenital heart disease with anatomical challenges.

## Data Availability

No data was generated to conduct this study.

## Research registration unique identifying number (UIN)

68041.
